# Lafora disease: a case report

**DOI:** 10.1186/s13256-022-03537-x

**Published:** 2022-10-03

**Authors:** Naim Zeka, Leonore Zogaj, Abdurrahim Gerguri, Ramush Bejiqi, Ragip Ratkoceri, Arlinda Maloku, Aferdita Mustafa, Labinot Shahini, Jeton Maxharaj

**Affiliations:** 1 Department of Neurology, Pediatric Clinic, University Clinical Center of Kosovo, Prishtina, 10000 Kosovo; 2 Department of Cardiology, Pediatric Clinic, University Clinical Center of Kosovo, Prishtina, 10000 Kosovo; 3 Institute of Pathology, University Clinical Center of Kosovo, Prishtina, 10000 Kosovo; 4 Aloka Hospital, Prishtina, 10000 Kosova; 5 University Clinical Center of Kosovo, Pediatric Clinic, Pristina, Kosovo

**Keywords:** Lafora disease, Inclusion bodies, Epilepsy

## Abstract

**Background:**

Lafora disease is a rare genetic disorder involving glycogen metabolism disorder. It is inherited by autosomal recessive pattern presenting as a progressive myoclonus epilepsy and neurologic deterioration beginning in adolescence. It is characterized by Lafora bodies in tissues such as brain, skin, muscle, and liver.

**Case presentation:**

We report a rare case of Lafora disease in a 16-year-old Albanian girl who presented at a tertiary health care center with generalized tonic–clonic seizures, eyelid twitches, hallucinations, headache, and cognitive dysfunction. She was initially treated for generalized epilepsy and received an antiepileptic drug. However, owing to resistance of seizures to this antiepileptic drug, a second drug was introduced. However, seizures continued despite compliance with therapy, and general neurological status began to deteriorate. The child began to have hallucinations and decline of cognitive function. She developed dysarthria and unsteady gait. When admitted to the hospital, blood tests and imaging examinations were planned. The blood tests were unremarkable. There was no relevant family history and no consanguinity. Electroencephalography showed multifocal discharges in both hemispheres, and brain magnetic resonance imaging revealed no abnormality. Axillary skin biopsy revealed inclusion bodies in apocrine glands. Consequently, the child was referred to an advanced center for genetic testing, which also confirmed diagnosis of Lafora disease with a positive mutation on *NHLRC1* gene.

**Conclusions:**

Even though rare as a condition, Lafora disease should be considered on differential diagnosis in progressive and drug-refractory epilepsy in adolescents, especially when followed by cognitive decline.

## Introduction

Lafora disease is a rare, genetic disorder of autosomal recessive inheritance characterized by presence of inclusion bodies (Lafora bodies) in the cells of heart, liver, muscle, and skin. It presents as a neurodegenerative disorder causing impairment of cerebral cortical neurons [1]. The disease usually manifests in previously healthy adolescents, and death commonly occurs within 10 years of symptom onset [2]. The two genes known to be involved in Lafora disease are *EPM2A* and *NHLCR1* [3]. Approximately 90% of cases of Lafora disease are caused by mutations in either the *EPM2A* or the *EPM2B* gene, which encode, respectively, a glycogen phosphatase called laforin and an E3 ubiquitin ligase called malin [4]. We report the case of a 16-year-old girl who presented with drug-resistant epilepsy and deteriorated neurologically over time, with headache, hallucinations, disorientation, dysarthria, and difficulty walking.

## Case presentation

A 16-year-old Albanian girl was referred to the Department of Neurology at the University Clinical Centre of Kosovo with seizures, twitches of the eyelids, hallucinations, and difficulty walking. She was the fourth child in the family and was born as a result of a full-term, well-controlled pregnancy. No history of drug use or exposure to radiation during pregnancy was reported. Delivery was completed at the Gynecological center at the University Clinical Center of Kosovo, with a birth weight (BW) of 3600 g. Cholecalciferol (D3) for prevention of vitamin D deficiency was administered regularly. She was vaccinated regularly as per national vaccination calendar. She was breastfed for 1 year and a half, and supplementary food was introduced at 6 months of life. Her growth and development were normal. No relevant family history was reported.

She was diagnosed with epilepsy 4 years ago. She initially presented with generalized tonic–clonic seizures, and antiepileptic therapy, valproic acid, was initiated by pediatric neurologist. Despite treatment and compliance with therapy, seizures continued; therefore, a second antiepileptic drug, levetiracetam, was introduced, and she continued to be on both antiepileptic drugs and comply with therapy. However, recently, she started to manifest hallucinations, emotional disturbances, and irritability, and the psychiatrist put her on fluoxetine. Later, she experienced cognitive decline, behavioral change, dysarthria, and difficulty walking. At admission, she weighed 40 kg and was disoriented, irritable, and unable to communicate. Physical examination of the musculoskeletal system revealed no erythema or swelling, but difficulty walking independently. Other system examination revealed no pathologic findings.

Overall, the results of routine blood tests showed normal values; hemoglobin (Hgb) was 11.8 g/dL, and platelet count was 156,000/mm^3^. The erythrocyte sedimentation rate was 5 mm/hr, and C-reactive protein was negative. Furthermore, liver enzymes, urea, and creatinine, as well as acid–base status of the blood, were within normal values. Other investigations, including urinalysis, were negative, too. Serum and cerebrospinal fluid analyses excluded infectious and immune-mediated etiology. Brain magnetic resonance imaging (MRI) and electroencephalography (EEG) were performed, and the psychiatrist was consulted.

Owing to progressive neurological status deterioration and lack of response to therapy, it was decided to send the child to a more advanced center abroad for genetic testing for Lafora disease because of the inability to perform genetic testing in the country. At the same time, axillary skin biopsy was performed and inclusion bodies (Lafora bodies) of apocrine glands were detected. The results of genetic testing also confirmed the diagnosis of Lafora disease, by detecting a mutation on the *NHLRC1* gene.

Therefore, parental testing to confirm their carrier status was recommended, as well as genetic counseling.

**Fig. 1:**
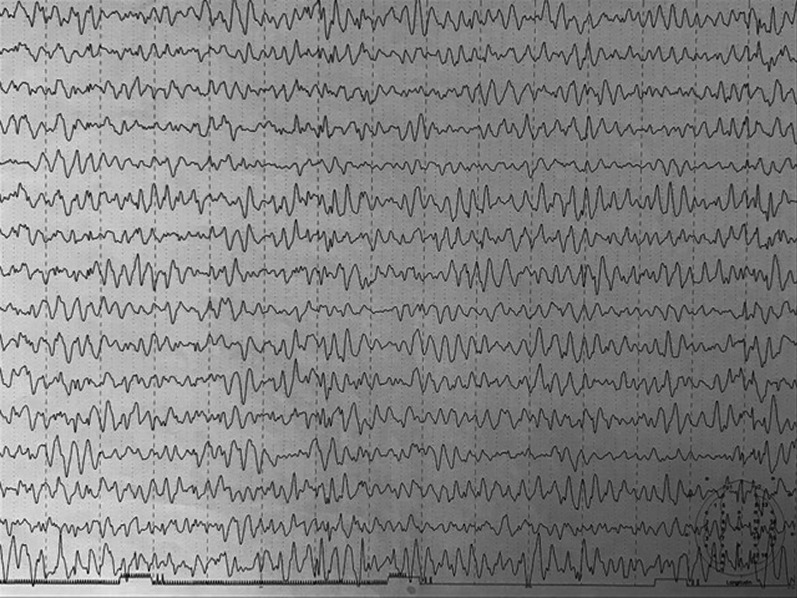
Initial Electroencephalogram (EEG) showing diffuse low voltage in both hemispheres

**Fig. 2:**
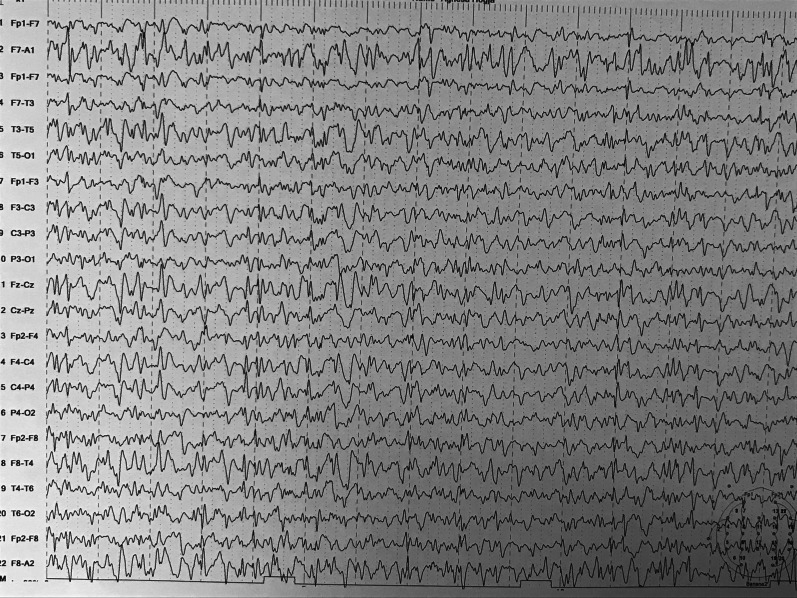
Follow-up Electroencephalogram (EEG) showing severe epileptic paroxysm activity in both hemispheres. Next, brain magnetic resonance imaging was planned (Fig. 3)

**Fig. 3:**
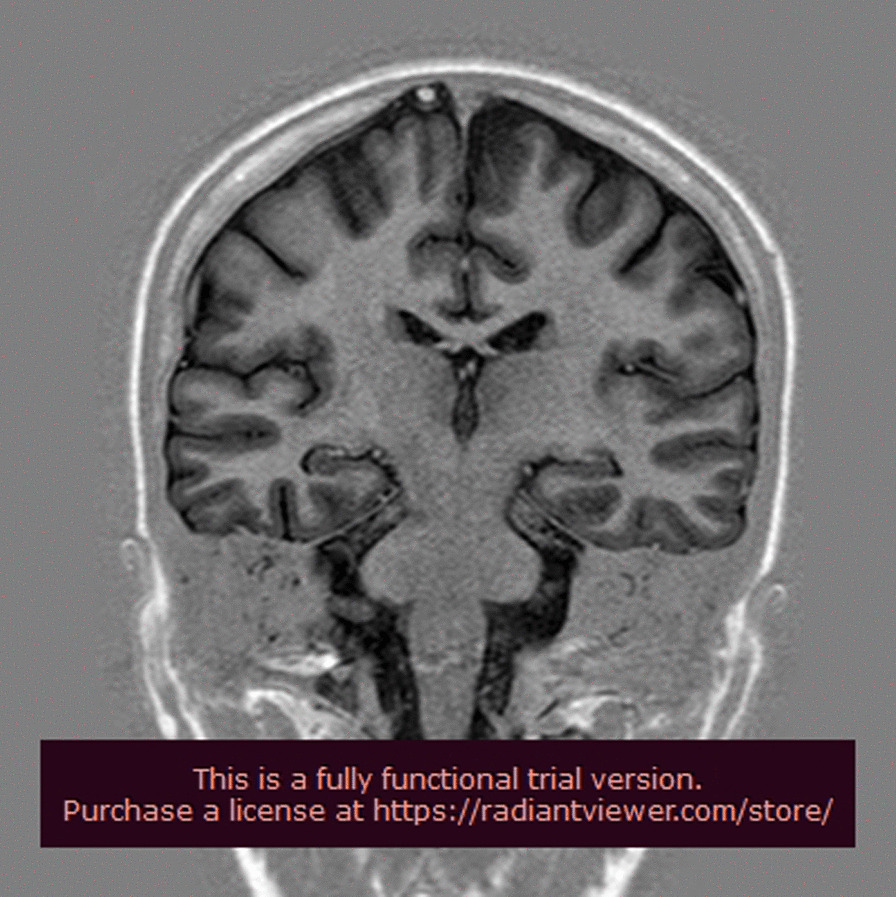
Brain magnetic resonance imaging showing normal findings

**Fig. 4:**
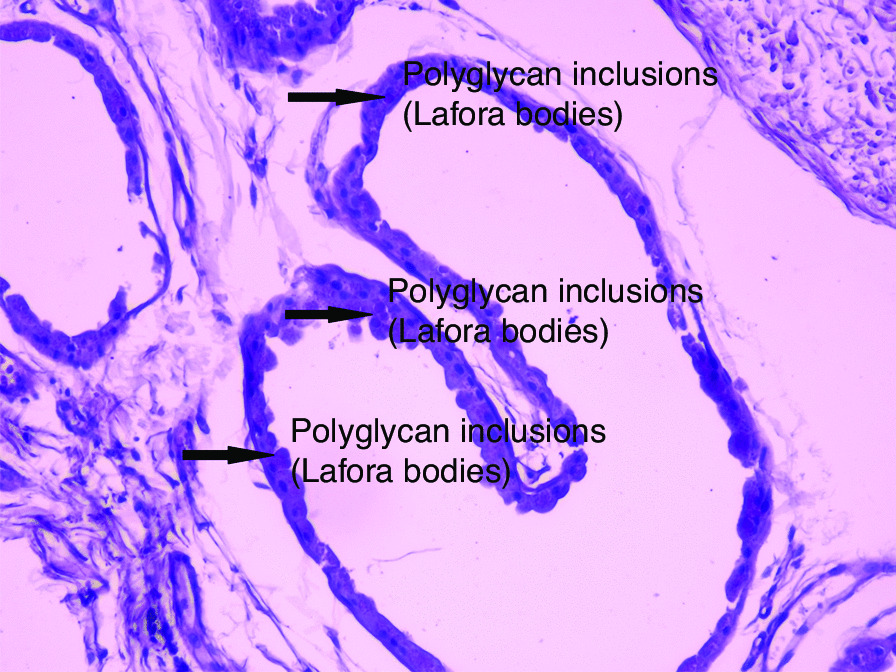
Axillary skin biopsy indicating myoepithelial cells containing polyglucosal (Lafora bodies) as well as chronic inflammatory infiltrates. The results of the biopsy reveal Periodic Acid-Schiff (PAS)^+^ polyglycan inclusions (Lafora bodies)

**Fig. 5:**
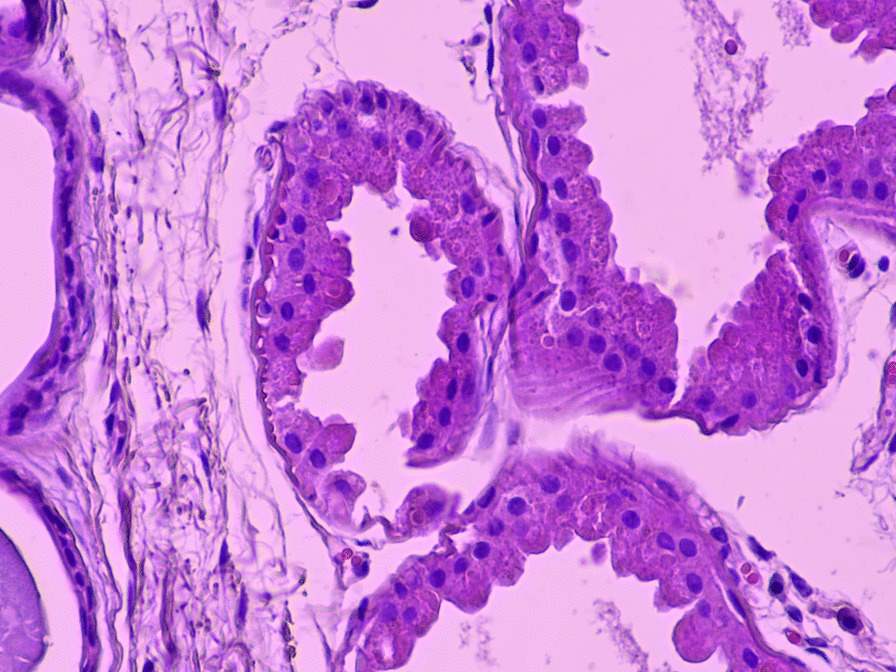
Axillary skin biopsy showing myoepithelial cells that contain polyglucosal (Lafora bodies) as well as chronic inflammatory infiltrates. The results of the biopsy reveal Periodic Acid-Schiff (PAS)^+^ polyglycan inclusions (Lafora bodies)

By the time the diagnosis was confirmed, the child had almost lost the ability to walk and speak. She could walk with great difficulty with the help of both parents on each side (the rest of the time via wheelchair) and could articulate a few sentences with difficulty. She still remains in this condition (Figs. 1, 2, 3, 4 and 5).

## Discussion

Progressive myoclonus epilepsy (PMEs) belongs to a group of inherited neurodegenerative disorders characterized by progressively worsening myoclonus and epilepsy, variable neurological dysfunction (ataxia, dementia), and possible associated signs and symptoms. LD is one of the main teenage-onset PMEs [6]. The first symptoms of LD appear during late childhood or adolescence (range 8–19 years; peak 14–16 years) [6]. The evolution of the disease is often fatal [7], with death commonly occurring within 10 years of symptom onset [2], most commonly due to pneumonia or complications related to degeneration of the nervous system [8].

Most patients are completely normal in childhood, with the exception of early learning difficulties in some. The early symptoms can include headache, decline in school performance, and convulsive seizures [9, 10]; however, it generally presents with tonic clonic seizures, followed by nonsynchronized, rapid, and massive myoclonic jerks in the extremity and mouth. Rapid progressive dementia and global cognitive dysfunction develop 2–6 years after disease onset [5]. Over time, those affected with Lafora disease have brain changes that cause confusion, speech difficulties, depression, decline in intellectual function, impaired judgement, and impaired memory. If areas of the cerebellum are affected by seizures, it is common to see problems with speech, coordination, and balance in patients with Lafora disease [1]. Currently, there is no efficacious treatment that controls the seizures and improves the cognitive decline in this disease [11]. Antiepileptic drugs (AEDs) are partially effective in the treatment of myoclonus and seizures but do not have a major influence on the progression of cognitive and behavioral symptoms [12, 13].

The diagnosis is based on detection of Lafora bodies on apocrine and eccrine sweat gland biopsies [5] and genetic testing.

Cranial images may be normal at the beginning, but with disease progression, diffuse atrophy may be seen; EEG is also normal at the beginning, with theta and delta activity appearing with the progression of the disease [5].

In our case, the clinical manifestations were typical, with generalized tonic–clonic seizures resistant to the initially administered antiepileptic drug, which progressed during the following years into cognitive decline, hallucinations, irritability, disorientation, and difficulty walking. By the time the diagnosis was made, the child had almost lost the ability to walk and speak. Although the child was referred for genetic testing for Lafora disease, skin biopsy of axillary glands was performed at our university clinical center. Lafora disease diagnosis can be made by detection of polyglucosan aggregates in myoepithelial cells surrounding sweat glands, also called Lafora bodies. However, distinguishing Lafora bodies from normal apocrine cell granules may be difficult, making genetic testing the preferred diagnostic method [14].

Mild brain atrophy has been described in 35–40% of patients with typical and mild Lafora disease, with normal MRI in the remaining patients [14]. EEG abnormalities often precede clinical symptoms and initially consist of almost normal or slowed background and generalized or focal paroxysmal activity [7]. Within a few years, a slowing of background activity becomes evident with frequent, superimposed bursts of diffuse epileptic discharges [7].

The main differential diagnosis concerns four other forms of PME: Unverricht–Lundborg disease (EPM1), neuronal ceroid lipofuscinoses, myoclonic epilepsy with ragged red fibers (MERRF), and sialidosis [7, 14].

A diagnosis of Lafora disease can cause severe psychological trauma to patients and their families, including but not limited to feelings of guilt and resentment, fear of younger siblings also becoming affected, and financial costs and concern regarding the availability of treatment options. Consequently, the diagnosis should be followed up with sustained, attentive genetic and psychological counseling and support [3].

In our case, the clinical status of the patient worsened over time as described in the literature, and she is now dependent on the wheelchair and unable to speak.

Lafora disease is a severe, autosomal recessive, progressive myoclonus epilepsy [3] with patients ultimately becoming completely disabled and bed-bound. Death usually occurs within 10 years from onset, often during status epilepticus with aspiration pneumonia [2, 7].

## Conclusion

Our case is a rare example of Lafora disease with onset of typical manifestations and progression of symptoms over years. Despite being a very rare condition, it should be considered on differential diagnosis in any child presenting with myoclonus epilepsy beginning in teenage years and progressing to cognitive decline and neurological deterioration in general.

## Data Availability

All of the data and materials will be available from the corresponding author upon request.

## Abbreviations

LD: Lafora disease
D3: Cholecalciferol
BW: Birth weight
MRI: Magnetic resonance imaging
Hgb: Hemoglobin
